# Rothmund-Thomson syndrome: Immuno-osseous challenges

**DOI:** 10.1186/1546-0096-9-S1-P312

**Published:** 2011-09-14

**Authors:** I Meyts, L De Somer, X Bossuyt, M-A Morren, K Devriendt, C Wouters

**Affiliations:** 1 Department of Pediatric Immunology, University Hospitals Leuven, Belgium; 2 Laboratory Medicine, University Hospitals Leuven, Belgium; 3 Department of Dermatology, University Hospitals Leuven, Belgium; 4 Center for Medical Genetics, University Hospitals Leuven, Belgium

## Background

Rothmund-Thomson (RTS) syndrome is a rare autosomal recessively inherited genodermatosis.. It is characterized by poikiloderma, small stature, skeletal and dental abnormalities, cataract, and an increased risk of cancer. The syndrome is caused by mutations in RECQL4 at 8q24.

## Aims

To describe the osseous and immunologic features of three patients with genetically confirmed RTS.

## Methods

Immunological investigation, x-ray imaging and bone densitometry were performed at time of the first visit to the combined rheumatology-immunology clinic.

## Results

All patients had characteristic poikiloderma as well as thumb anomalies. They were born dysmaturely and presented with failure to thrive.

Age at genetic diagnosis was 5y, 4y and 3y for P1, P2, P3.

Osteopenia and abnormal metaphyseal trabeculation of bones were striking on the initial skeletal survey in all patients. Z-scores on DXA scan were -0.1, -1.1 and -1.2 for P1, P2, P3 respectively at presentation. The presentation in P2 was dramatic with 6 fractures in upper and lower extremities and subluxation of both radii.

All patients were suffering from recurrent chest infections. P1 had granulomatous skin inflammation following primo VZV infection. All patients have low switched memory B cells for age, P1 has IgG2 deficiency. P1 and P3 have IgM deficiency. P1 and P3 have specific polysaccharide antibody deficiency. Results are pending for P2. All receive prophylactic antibiotics. P1 is treated with subcutaneous immunoglobulin substitution.

## Conclusion

RTS is a genodermatosis with variable clinical presentation and course. Our observation of severe bone abnormalities and associated immunodeficiency merits attention for optimal management of these patients.

